# Why should HCWs receive priority access to vaccines in a pandemic?

**DOI:** 10.1186/s12910-021-00650-2

**Published:** 2021-06-27

**Authors:** Xavier Symons, Steve Matthews, Bernadette Tobin

**Affiliations:** 1 Plunkett Centre for Ethics, Australian Catholic University, 7 Ice Street, Darlinghurst, NSW 2010 Australia; 2 Thomas More Law School, Level 7, 486 Albert Street, East Melbourne, 3002 Australia

**Keywords:** COVID-19, Vaccines, Healthcare resource allocation, Reciprocity, Utilitarianism, Deontology, Policy

## Abstract

**Background:**

Viral pandemics present a range of ethical challenges for policy makers, not the least among which are difficult decisions about how to allocate scarce healthcare resources. One important question is whether healthcare workers (HCWs) should receive priority access to a vaccine in the event that an effective vaccine becomes available. This question is especially relevant in the coronavirus pandemic with governments and health authorities currently facing questions of distribution of COVID-19 vaccines.

**Main text:**

In this article, we critically evaluate the most common ethical arguments for granting healthcare workers priority access to a vaccine. We review the existing literature on this topic, and analyse both deontological and utilitarian arguments in favour of HCW prioritisation. For illustrative purposes, we focus in particular on the distribution of a COVID-19 vaccine. We also explore some practical complexities attendant on arguments in favour of HCW prioritisation.

**Conclusions:**

We argue that there are deontological and utilitarian cases for prioritising HCWs. Indeed, the widely held view that we should prioritise HCWs represents an example of ethical convergence. Complexities arise, however, when considering who should be included in the category of HCW, and who else should receive priority in addition to HCWs.

## Background

In viral pandemics policymakers face difficult ethical decisions over the allocation of scarce healthcare resources, giving rise to debate over the correct policy and its ethical grounding. Our aim in this debate is to present the case for ethical convergence in support of the position that healthcare workers (HCWs) should receive priority access to vaccines. This question is especially relevant in the coronavirus pandemic with governments and health authorities currently facing questions of distribution of COVID-19 vaccines.

## Introduction

Many ethicists argue that healthcare workers (HCWs) should receive priority access to a vaccine in an influenza pandemic [1–6]. Yet differing ethical justifications are offered as to *why* this is so. In this article, we identify and articulate the most commonly presented arguments in favour of prioritising HCWs for access to a vaccine in a pandemic [7].^1^ For argument’s sake, we focus on the COVID-19 pandemic, though we believe that the main thesis of this paper has application in pandemic situations of relevant similarity. We argue that there are deontological as well as utilitarian cases for prioritising HCWs [8].^2^^,^^3^ Indeed, the widely held view that we should prioritise HCWs represents an example of *convergence of different and (on occasion) conflicting ethical theories*. We defend this convergence by appealing to the claim first that policymakers should make predictions about what happens in a pandemic based on a relatively stable evidence base, and second that the nature of policy itself takes time, for example, given the required public messaging and the mobilization of infrastructure. Finally, we address some complexities which arise when considering who should be included in the category of HCW, and who else should receive priority in addition to HCWs.

In “Background” section, we discuss the impact of the coronavirus pandemic, proffer a definition of the term ‘healthcare worker’, discuss extant COVID-19 vaccines, and make explicit the epidemiological and policy assumptions that our argument relies on. In “HCWs and pandemic rationing: a literature review” section, we discuss the existing literature on the rationing of this particular medical resource in a pandemic. In “Deontological and utilitarian arguments in favour of prioritising HCWs” section, to support our claim of convergence we canvas several versions of deontological and utilitarian arguments in favour of prioritising HCWs; finally, we show how such convergence provides decision-makers with reassurance and confidence for community engagement. This requires transparency and the presentation of good reasons to the public especially where emergency protocols for distribution of a vaccine are triggered.

## Background

### The coronavirus pandemic

The coronavirus pandemic is arguably one of the most serious public health challenges that the world has faced in the past century. At time of writing (June 2021), there have been over 175 million cases of COVID-19 and approximately 3.75 million deaths reported worldwide [9]. While the impact of the virus has been particularly bad in countries such as the United States, India, Brazil and Russia, every nation has been severely affected—either directly by the virus itself or indirectly by the social and economic effects that it has wrought on societies. One research team has estimated that the COVID-19 pandemic cost more in 2020 than the world’s combined natural disasters in any of the past 20 years [10].

Particular social groups and professions have been disproportionately affected by the virus. Older members of the community are known to be especially vulnerable to severe illness if they contract COVID-19, and in many countries a disproportionate number of COVID-19-related deaths has been either in or linked to aged care homes [11]. Social and ethnic minorities also face a higher risk of contracting COVID-19 and developing serious illness. The coronavirus pandemic has, for example, been particularly devastating for the African American community in the United States. African American individuals are over two-and-half times more likely to contract COVID-19, five times more likely to require hospitalisation, and two times more likely to die from the virus [12].

### Defining Healthcare workers

The thesis of this paper is that HCWs ought to be given vaccination priority, so we should outline at this early stage how we will understand this category. It turns out that defining the set of all and only healthcare workers is a non-trivial task, and though it is beyond the scope of this paper to address a full range of qualifications, we will restrict ourselves to a commonly held and intuitively plausible categorization. We should note first that the set is wider than healthcare *professionals (those who fall under a regulated body)*, yet not so wide as to include workers who (say) accidentally, or incidentally, find themselves working in a healthcare setting. So, though we think that in borderline cases the default should be to include rather than exclude, we would, for example, exclude from the category a plumber who has been called out to fix a broken pipe in a hospital. Our definition includes:

Individuals who (1) deliver care and services *directly* in a way that aims to heal the sick or restore human health, or (2) deliver care and services with this goal *indirectly*.

The Centre for Disease Control and Prevention includes the following types of workers: physicians, nurses, emergency medical personnel, dental professionals and students, medical and nursing students, laboratory technicians, pharmacists, hospital volunteers, and administrative staff.^4^ The difficulty arises in saying which groups fall under (2). For instance, is a regularly contracted driver for a medical waste disposal company to be included? We think the answer is ‘yes’, and that is because this person is in regular employment fulfilling the role, and a lack of this service would impact, at least to some extent, the goal of healthcare built into the definition.

Based on our definition above, *ceteris paribus*, healthcare workers delivering care directly to patients with the virus (because they have the virus), should receive first priority; healthcare workers delivering care directly to patients who might, for all they know, have the virus, should receive second priority; and healthcare workers who fall under (2) should receive third priority. The ‘*ceteris paribus*’ qualification simply acknowledges the possibility, as stated earlier, that these normative judgements are based on general predictions only. For instance, it is well known that the poor, the elderly in residential care, or those forced by circumstances to congregate in close proximity such as those in the prison population, are much more vulnerable to outbreaks which might require ad hoc emergency measures in which they gain temporary priority to vaccination.

The impact of COVID-19 on HCWs is well-documented. Frontline HCWs are significantly more likely to contract the virus on account of their close-proximity to, and/or their contact with, coronavirus patients. Once infected, they are likely to spread it to their colleagues. The risk of contracting coronavirus is compounded where HCWs have inadequate access to personal protective equipment. A 2020 *Lancet Public Health* study conducted using US and UK data found that “front-line health-care workers had at least a threefold increased risk of reporting a positive COVID-19 test and predicted COVID-19 infection, compared with the general community, even after accounting for other risk factors” [13]. Unsurprisingly, the study also found that “reuse of PPE or inadequate PPE were… associated with a subsequent increased risk of COVID-19” [14].

### COVID-19 vaccines

At the time of writing (June 2021), several COVID-19 vaccines have received emergency, provisional or full regulatory approval and are being rapidly distributed to priority population groups across the globe. Vaccines such as the Pfizer and Moderna vaccines would appear to have efficacy rates of up to 95% in preventing symptomatic disease, while the Oxford AstraZeneca vaccine is approximately 76% effect in preventing symptomatic COVID-19 [14]. Several countries such as the United States, Israel and the United Kingdom have already vaccinated a significant proportion of their population. These countries have witnessed a sharp drop in case numbers and hospitalisations and deaths from COVID-19. The COVAX Facility—an initiative led by Coalition for Epidemic Preparedness Innovations (CEPI), Gavi and the World Health Organization (WHO)—has also helped ensure some degree of access to vaccines for developing nations, albeit with less success than was originally anticipated.

There is extensive speculation about the relative safety and efficacy of different approved vaccines. The AstraZeneca and Janssen COVID-19 vaccines, for example, have been linked to a rare clotting condition called ‘vaccine induced prothrombotic immune thrombocytopenia’ (VIPIT), whereby a very small number of vaccine recipients develop venous or arterial blood clots (often near the brain) combined with a low platelet count [15]. Earlier estimates suggest that the condition may affect approximately one out of every 100,000 vaccinated individuals (mostly women under the age of 50), with a fatal rate of roughly one in 10 [16]. With this in mind, several regulatory agencies have limited access to the vaccine to people of a certain age (e.g., people 50 and older in Australia).

We will leave to one side questions of whether one vaccine is either more safe or more efficacious than another. We would note, however, that this may provide nuance to the question under consideration, viz*.*, who should receive a vaccine first. If indeed one vaccine were demonstrably more effective and/or safer than another, then perhaps a case could be made for making that vaccine available to priority groups first. We will presume, however, that there is parity between the different vaccines available.

### Ethical convergence, changing circumstances, and ethical policies

The position we defend here trades on the assumption that the difficult decisions arising in a viral pandemic over distributing a vaccine arise at the contextual level of specific questions over *scope issues* (who falls under the extension ‘HCW’), and *implementation* (e.g., community engagement). But the answer to the general question concerning which of the major ethical theories may be marshalled in these decisions, we claim, is clear: *both* point in the same direction, towards the prioritization of HCWs. In one respect this convergence is not the least surprising—different moral theories prescribe identical courses of action on many issues. In another respect it is surprising given this core conflict: for utilitarians the right-making properties of actions depend only on their consequences; but for deontologists the right-making properties of actions are defined not only with regard for their consequences. Now if we considered a strict version of utilitarianism, such as act-utilitarianism—where registering the consequences of each particular act is required in order to ascertain its rightness—the convergence we are positing could not be seriously entertained. As is sometimes said, such versions are hostage to empirical fortune, and in this context, policymaking would be at the mercy of an ever-changing empirical situation. This would prove disastrous because, as we will argue, it could not be operationalised by regulators who require a unified, and stable, set of rules that allow uptake by an uncertain public. To make policy, against utilitarian standards, then, the version of the theory needed is a rule-based version in which the rules being set by policymakers may rationally advert to a best consequences principle. Such a principle *could* converge with that of the deontologist, as we now explain.

We begin by pointing out that the application of any ethical theory in the context of a viral pandemic must be such as to articulate with real world policymaking, and policymaking decisions require (1) appropriate generalizations that abstract from the social environment in which those decisions are made, and (2) a degree of bureaucratic inertia and stability in order to be effective—chopping and changing rules is self-undermining. Let us explain these two elements in turn.

First, then, policymakers in a pandemic must make ethically informed decisions based on general predictions about the course of a pandemic, given their understanding of the nature of the virus, the state of the world at a time *t*, and a prediction about the course of the pandemic post-*t*, given the public health response. This evidence combines conceptualizations related to which measures to pay attention to, such as the reproduction number of the virus, and the efficacy of a vaccine, along with registration and identification of empirical realities such as how many people have contracted the virus at a given time. We do not downplay the extraordinary complexity of these predictions, including the range of variables and the levels of uncertainty. Nevertheless, the nature of the evidence base—what evidence we need, and why we need it—for decision-making here are well known.^5^ Thus there is a set of empirical conditions, and required concepts, associated with viral pandemics that typically underpins what is needed to justify a policy supporting the vaccination of HCWs.

The second element is that policymakers must make ethically informed decisions so that they *translate into policy*, and it is a conceptual truth about policies that their implementation occurs over time and with unified persistent purpose. Policies can provide signals to the public that planning is being taken seriously, and this requires stable enduring forward-looking public commitments. A way to put this is that a policymaker (in a pandemic) must be constrained in sophisticated ways to act according to rule-based ethical principles that take time. For example, public messaging depends on it, and setting up and paying for infrastructure to deliver vaccines requires extensive planning, and resources. This is not the kind of stance that, for example, an act utilitarian can adopt, so the form a utilitarian theory takes in this context must adhere to some rule-oriented stance, one that aims at the minimization of disutility via extended policies constructed for that purpose. Our wider point though is a conceptual one about the nature of a policy and its institution over a required length of time. Having this understanding ought then to feedback into one’s theoretical stance, and though policy can, and should, respond to changing circumstances, there are reasons why it must also move slowly enough for its aims to be taken up by those who give effect to it; else there is no point in having a *policy*. In this regard, policy must take account of the consequences of a pandemic situation at the sweet spot between predictive understandings of viral behaviour in a population together with planning for exceptions to those predictions. (Consider, as an example, viral mutations with the potential to be drug-resistant to extant vaccines.) That is, it must take account of the first element above: policy must respond to an understanding of the set of empirical realities characterizing a viral pandemic.

We are not asserting that viral pandemics have typical well understood *inevitable* trajectories, only that policymakers rationally ought to presuppose that the levers of public policy at their disposal should have predictable effects, given their understanding of the behaviour of the virus in a population. Some remarkable examples include the successes of lockdowns in New Zealand and some Australian states in eliminating the virus prior to the availability of a vaccine. These successes were possible because experts understood what was required and policymakers gave effect to their prescriptions. This understanding was based on an evidence base that included the reproduction number (a contagion measure), prevalence levels at *t*, predicted rates of morbidity and mortality under varying conditions of care, and disease profile (e.g., length of time from infection to symptoms of clinical significance). The availability of vaccines adds to this evidence, including the number and type available, logistical considerations around distribution, and their efficacy (as determined by trials) and effectiveness (real-world setting results). Importantly, as mentioned above, the potential for viral mutations under varying conditions and timeframes is required. Finally, the ways each of these measures relate to each other in conjunction with mitigation measures such as lockdowns must be presupposed as part of the first element listed above.

To summarise, we are claiming that the context of a viral pandemic requires policymakers to incorporate an accurate assessment of the empirical conditions, to be cognizant of changing circumstances and to flexibly adapt where needed, but this knowledge has limits. Given these knowledge limits, policymakers are forced to make ideal predictions. Our claim is that these ideal predictions are such that both deontology and consequentialism can be marshalled to support giving priority to HCWs. Such agreement is good news for many reasons, but an important, often unnoticed, feature of convergence is that it disarms potential controversies over the lexical ordering of principles. If one acts according to one of them, one thereby acts according to the other; and vice versa.

We do note that policymaker decision-making *ideally* should rely on the total time to vaccinate, and include the total population within a jurisdiction.^6^ A short time span to vaccinate an entire country—say over two months or so—*might* affect our claim that HCWs are given priority; perhaps the elderly should be vaccinated first in this scenario. But the point is, such examples fall outside the situation as we are positing it, based on a viral pandemic like COVID-19.^7^

In the next section, we will consider how a vaccine ought to be distributed, and whether, in particular, HCWs ought to be prioritised.

## HCWs and pandemic rationing: a literature review

In this section, we review the existing literature on HCWs and the rationing of vaccines in a pandemic. We argue that there is a broad consensus that at least some HCWs should receive priority access to a vaccine during a pandemic. This consensus takes two forms—a consensus of scholarly opinion, and a convergence of rival ethical approaches. First, there is a broad scholarly consensus that frontline HCWs should receive priority access to a vaccine [1–6]. Indeed, a recent critical-interpretative review of the literature by Williams and Dawson found that “the most commonly justified group given priority [in an influenza pandemic] was HCWs” (Williams and Dawson 2020, 3) [17]. Theorists who have written specifically about rationing during COVID-19 pandemic have arrived at the same conclusion [18, 19].

Additionally, what we see in the literature is agreement among competing ethical approaches to pandemic rationing. Very different normative approaches (both deontological and utilitarian) support the conclusion that HCWs should be prioritised. In addition, a ‘deliberative democracy’ approach, that is, an approach based on community attitudes, is supportive of HCW prioritisation. The data on community attitudes to healthcare resource prioritisation suggests that the public would support the prioritisation of HCWs [13, 20].

As stated earlier, there is, admittedly, disagreement in the literature about who counts as a HCW.^8^ Different authors circumscribe this category differently, and as Williams and Dawson point out, the nature and size of groups varies based on professional status and proximity to pandemic-affected patients. They, and others, point out there is also support for vaccine priority for ancillary occupational groups including ‘vaccine manufacturers, emergency services workers, and those working in basic infrastructure such as utility, transport, policing, food manufacturing and distribution, and communications’ [3].^9^ Indeed some of the arguments in favour of prioritising HCWs presuppose that they will be instrumental in supporting society’s response to the pandemic (frontline responders), whereas others apply to HCWs as such—independent of their role in the pandemic response.

There is another reason why the definition of a HCW is relevant to judgements about healthcare prioritisation: a broad definition could be taken to imply that the providers of other related essential services, such as those in transport, supply, law and order and so on, also deserve priority. It follows from this, that on a broader definition of HCW, utilitarian arguments for vaccine prioritisation gain more traction than deontological arguments because the former is concerned primarily with questions of efficiency, whereas deontological arguments include a normative requirement to compensate the HCW who has made sacrifices, and taken risks, in the service of pandemic control which do not obtain in the case of these wider occupations. Our position is that those who fall into the highest priority category are HCWs who directly care for people with the virus, where compensation and efficiency arguments both apply. For those outside this category, the criteria for inclusion would scale accordingly, depending on professional status and proximity and risk. Thus a healthcare administrator, for example, might be a moderate risk for catching and spreading the virus, but is not owed anything more special than an administrator in a setting that is unrelated to healthcare.

## Deontological and utilitarian arguments in favour of prioritising HCWs

In this section, we explicate the deontological and utilitarian arguments that underpin the claim for giving HCWs priority access to vaccines in a pandemic.

### Deontological-style justifications for HCW prioritisation

A first form of argument is deontological, more particularly an argument that prioritises HCWs because of a debt they are owed. This form of argument may be characterized in different ways.^10^ First, a debt may be owed because the person, or group, *deserves* a reward on account of something done in the past [21].^11^ Second, it may be owed because of a real, or tacitly understood, *contract*, that in providing a service, the one who receives that service (again, a person, group, or in this case an entire society) is indebted to the service-giver and thereby has special reasons to offer reward because of it. (And, closely related to this purely contractual idea is the idea that discharging an obligation to pay off this debt is a matter of compensating the person, or group, for burdens incurred.) [22].^12^ In what follows, we spell out the deontological case for priority access with reference to these distinctions.

Based on the desert-based characterisation above, we have an obligation, as a matter of justice, to return good for good. A society should compensate HCWs for the *sacrifices* they have made and the *risks* that they have accepted in providing protection and care for the community in the face of a pandemic. On this view, people ought not reap the benefits of social cooperation without contributing a fair share to producing those benefits.

It is worth spelling out what sacrifices HCWs make and why these are relevant to the moral argument. First, healing the sick is *morally meritorious*, and—to offer two plausible analogues—just as we should reward those students who study hard, and just as we should properly remunerate rescue workers who place themselves in harm’s way, so too should we reward professionals who provide care for the sick. Second, HCWs have spent a lot of *time and resources* preparing themselves for their specialised role—they have studied hard, and made sacrifices. And third, that for which they have made these sacrifices (entry into the profession of healthcare) has as its point not simply a personal benefit but something of *significant social value*. According to this argument, frontline HCWs deserve a benefit in light of the sacrifices made in pursuing and fulfilling their professional role.

In considering the non-backward-looking (purely) contractualist dimension of these argument types we focus on the risks HCWs take in the course of their professional duty. In a pandemic the specific risk associated with the reciprocal duty to prioritise HCWs is of course the increased risk of infection taken on as part of their work. As Verweij writes:

If there is an increased risk to health care workers, then reciprocity supports giving priority access to protection and treatment in such a way that their risk will be similar to that of other citizens. (p.165) [3]

Now a critic might object to this argument by pointing out that it is part of the job for HCWs to take these sorts of risks (‘this is what they signed up for’).^13^ But that fails to distinguish the risks inherent in the profession that arise in the normal course of one’s occupation, compared with risks that fall outside this normal course. Plausibly healthcare work in a pandemic of the magnitude and severity of COVID-19 is not something that HCWs could be reasonably expected to foresee when they joined their respective professions. Admittedly, this is a matter of degree, but nevertheless, the history of pandemics comparable to COVID-19 reveals a justificatory infrequency: since the 1918 influenza pandemic there have been only four comparable pandemics, including the H2N2 strain of Flu (1957), the H3N2 strain of Flu (1968), the H1N1 strain (2009), and the HIV/AIDs virus from the early 1980s. A HCW versed in this history might justifiably estimate its occurrence to be a once-in-a-career event.

A critic might also object that there is a problem with reciprocity arguments insofar as we seek to set a principled limit on their scope. As McGuire, Ausilio and Davis et al.write:

If HCWs ought to be prioritized, why not these other groups as well? If not these, then why HCWs? A question is raised as to whether the reciprocity owed is due to risk taken or life-saving services rendered. In the latter case, first responders and bedside HCWs would arguably have higher priority than cashiers, but this is controversial. (p.18) [14]

The question, in other words, is whether we have an obligation based on reciprocity to prioritise essential service workers in general, and not just HCWs. We do not provide a detailed answer to this question due to the limited scope of this article. Suffice to say that other essential service workers should also be prioritised if empirical evidence suggests that they face a similar risk of contracting the virus. Justice requires that we treat like cases alike, and we would argue that the arguments already raised might well apply to essential service workers if the conditions we cited for HCWs also obtain. Alternatively, one might argue that there is something special about the healing professions that makes them worthy of priority. Yet it seems to us that purely contractualist (especially risk-based) arguments in favour of prioritising healthcare professionals can be extended to essential service professions generally. That might have settled the issue of priority, and suggest that the category of HCW is too narrow, but the utilitarian-style justifications for HCW prioritisation make the difference. We now turn to those.

### Utilitarian-style justifications for HCW prioritisation

Utilitarian arguments are based on the idea that our actions ought to aim at the maximization of utility.^14^ Utility has been variously defined by philosophers as happiness, pleasure, well-being and preference satisfaction. In healthcare contexts, however, ethicists typically define it in terms of concrete health and social outcomes. These include indices such as patient survival and quality of life, as well as the broader economic and social impacts of the provision of prophylaxis or treatment for population groups. Within this context, then, it might be argued on utilitarian grounds that we should give priority to HCWs because doing so will lead to less harm (or greater benefits) than would otherwise be generated on any other salient course of action. This general claim gets expression in a range of specific practical considerations that relate to the pandemic context.

First, there is the question of *efficiency* in stopping the spread of a virus, given that it is frontline HCWs who need to be available to care for the sick and thus minimise morbidity and mortality. Prioritising HCWs will allow us to maximise aggregate population health, particularly in a pandemic.^15^ Related to this, those HCWs who are directly responsible for rolling out the vaccine themselves should be among the first to receive a vaccine. That is, since HCWs can infect patients, their own loved ones, and members of the community should they contract COVID-19, we should prioritise their access to a vaccine based on both their higher risk of infection as well as the higher likelihood that they will become ‘super-spreaders’.

Second, prioritising HCWs for a vaccine has the effect of ensuring confidence in the general citizenry—including HCWs—with respect to the healthcare system. HCWs will feel more confident of their safety in working with others, and patients will feel safer in coming to hospital. This is not a negligible point, considering the mounting evidence that fewer patients have been attending hospitals in the COVID-19 pandemic due to safety concerns [23].

Does this argument presuppose the prioritisation of HCWs based on their social value? Does it open the door for socially loaded judgments about other groups in society who it might be argued should receive priority access to healthcare resources? As McGuire, Ausilio and Davis et al. write,

Prioritizing HCWs for their instrumental value in the fight against COVID-19, however, involves an assessment of their relative social value. These assessments, while problematic in themselves, are notoriously susceptible to hidden biases and prejudices that may further exacerbate existing health, racial, and social disparities. (18) [14]

The claim that the rationale here disguises biases in favour of HCWs as a special group need not of itself bother the utilitarian. It becomes morally problematic on utilitarian grounds only when it can be shown that such biases lead to diminishing social utility. Moreover, the utilitarian may claim that in so far as the pandemic context is concerned—a relatively rare one-off event—the issue of biases will not manifest as a wider problem outside the pandemic emergency conditions. In any case, there is no contradiction in claiming that such biases may be addressed simultaneously with the emergency response to the pandemic in which HCWs are prioritised for the vaccine. Indeed, there are existing influenza vaccine distribution plans that profess to satisfy both these desiderata (i.e., they both prioritise HCWs *and* avoid socially loaded judgements that put minorities and vulnerable populations at a disadvantage.) [24].

### Implementation and community engagement

The arguments so far demonstrate an ethical convergence in concluding that HCWs have priority when receiving a vaccine in a pandemic. Such convergence provides decision-makers—in government or hospital administration—as well as clinical and related staff, with some reassurance about the right way forward should we reach a point with COVID-19 where emergency protocols for distribution of the vaccine are triggered. Having a robust consensus among decision-makers in relation to the ethical foundations for political and policy decisions on this question is critical, but the implementation of these decisions requires community engagement.

It is important, then, that health authorities seek where possible to engage relevant community stakeholders in their decision-making processes. McGuire, Ausilio and Davis et al., for example, note:

...community engagement strategies can exemplify the principle of respect for persons in community and thereby engender and promote mutual trust and shared accountability between [HCWs], their patients, and communities. In the unprecedented crucible of today’s COVID-19 pandemic, these goods, principles, duties, and values will be put to the ultimate test. (21) [14]

At the very least, health authorities should be transparent with the community about the reasons why they have chosen to give HCWs priority access to a vaccine [25]. The process of community engagement is an important part of providing a robust justification for prioritising HCWs in a pandemic.

## Conclusion

There is widespread agreement that HCWs should receive priority access to a vaccine during an influenza pandemic, and, indeed, such a sentiment is echoed in the literature on resource allocation in the coronavirus pandemic. Different reasons are, however, offered for this conclusion—and many of these reasons are drawn from rival ethical frameworks. In this article, we have analysed the justification that is offered for HCW prioritisation, and have considered what implications competing justifications might have for real-world vaccine rationing. We have identified the importance of the translation of ethical principles into policy, and what that means in the light of changing empirical conditions: policymaking presupposes limits to the type and degree of adaptation possible. We have provided an overview of the *ethical convergence* between rival normative approaches on the question of HCW vaccine prioritisation. We have responded to a series of objections that could be made to the prioritisation of HCWs on the basis of reciprocity or utilitarian considerations. We have noted an ambiguity in the literature concerning which HCWs should be prioritised, as well as whether other essential service workers, in addition to HCWs, should receive priority. Lastly, we adverted to the importance of community consultation when developing any ethical framework for healthcare resource allocation. We recommend that health authorities keep these considerations in view as they seek to develop a just and equitable framework for COVID-19 vaccine allocation.

## Data Availability

Not applicable.

## Abbreviations

CEPI: Coalition for Epidemic Preparedness Innovations
COVAX: The COVID-19 Vaccines Global Access Facility
COVID-19: Severe acute respiratory syndrome coronavirus 2 (SARS-CoV-2)
H1N1/ H2N2/ H3N2: Subtypes of the influenza A virus
HCWs: Healthcare workers
HIV/AIDS: Human immunodeficiency virus/acquired immunodeficiency syndrome
WHO: World Health Organisation
VIPIT: Vaccine induced prothrombotic immune thrombocytopenia

## Footnotes

1 Separate arguments are required for prioritization of non-vaccine healthcare resourcing, though there are strong cases for such things as priority testing, See Black, J. et al. [7].

2 Nevertheless, this is a narrow convergence of principles within western liberal democratic polities. Wide convergence is unlikely; see Arras, J. [8].

3 We acknowledge rarer egalitarian-style arguments in which lotteries or even first-come-first-served systems are proffered as solutions to vaccine scarcity. But these approaches are typically thought unfair, and certainly the latter looks grossly inefficient. Another line on this, however, is that the upshot to prioritizing HCWs is in fact a more equal valuing of life. See Emanuel E, Wertheimer A. [4]

4 See Recommended Vaccines for Healthcare Workers|CDC.

5 See King, N. Briefing: Evidence and Uncertainty During the COVID-19 Pandemic. Briefing: Evidence and Uncertainty During the COVID-19 Pandemic|Max Bell School of Public Policy—McGill University.

6 What counts as a jurisdiction is currently debated: at one extreme end is pure nationalism (where only the citizen’s interests of a particular country are morally considered); at the other extreme is pure cosmopolitanism (where country-of-residence is morally irrelevant). See Schaefer, O and Savulescu, J. Vaccine Nationalism: striking the balance. Practical Ethics: Ethics in the News. https://blog.practicalethics.ox.ac.uk/2021/04/vaccine-nationalism-striking-the-balance/.

7 We thank an anonymous referee for asking us to address this specific point and example.

8 Op cit. footnote 4.

9 See Verweij, M. [3], p.3.

10 We thank an anonymous referee for suggesting we clarify the various grounds that underpin our expression of the deontological argument we outline here.

11 See, for example, Persad et al. [21]. See p. 426 for their discussion of the reciprocity principle.

12 See Tom Walker [22]. On p. 210 Walker points to a contractual dimension in the case of a rescuer (such as a fire-fighter) to forestall an argument that they do not deserve equal treatment because of risks undertaken as part of their job.

13 Tom Walker [22], p.209 also discusses this ‘knowing the risks’ point.

14 Utilitarians, however, differ on whether the maximisation of utility is the only moral principle, or simply one among many. Disputes occur over the nature of utility; over whether to maximise over lifetimes or phases of lifetimes, or even mere experiences; and over whether everyone’s happiness (or utility) is of equal worth.

15 Op cit. footnote 4.
